# Supplementary material for: Resilience in Older People: A Concept Analysis
Source: Healthcare (Basel). 2023 Sep 8;11(18):2491. doi: 10.3390/healthcare11182491 (PMC10531380; doi:10.3390/healthcare11182491)
Supplement: Supplementary file 1 [file healthcare-11-02491-s001.zip › healthcare-2577416-supplementary.pdf]

Table S1. Included studies and the attributes, antecedents, consequents and empirical elements of concept analysis

ELEMENTS	CHARACTERISTICS/CODE	LITERATURE DESCRIPTION
ATTRIBUTES	Resources available<br>**A3, D3, E1, F1, I1, R1, R14, H3, R22, S3**	Environmental Capital; Cultural Capital; Economic Capital; Social Capital; Social Relationships; Family and Friends; Love and Friendship; Having Divine Support; Faith in God; Spirituality.
	Positive behaviors<br>**B2, B3, E2, I1, N1, M4, P6, P7, R1, R14, H3, E5, D4, D6, A9, C6, S4**	Courage and Strength; Resistance; Hardiness; Positive sense of self and an optimistic outlook on life; Strong positivity demonstrated by identity projects, redemptive sequences and narrative openness; Positive comparison with others; Interpersonal Control; Mastery; Belief in self; Competence; Maintaining purpose; Determination; Sense of purpose; Self-determination; Strives toward goal achievement; Prosocial Behavior; Expressing Gratitude; Sense of Humor; Ability to use humor; Problem-solving skills; Meaningful work and activities; Flexibility; Creativity; Autonomy; Recovery; Sustainability; Warrior; Adaptative; Moving-On; Self-esteem; Esteem; Strong self-efficacy.
ANTECEDENTS	Sociodemographic Characteristic<br>**B1, C5, D1, D2, E3, I1, M3, P4, P8, R2, R3, R9, R10, T1, A9, D4, E6, M5, R17, R20, S2, S3, T6**	age (older people > 65 years old, including 80 and over), gender (female and male), education (higher), income (higher) and status marital (have a partner)
	Experiences of Adversity<br>**A3, B2, B3, B4, C2, C3, E3, F1, M2, M3, N2, M4, P3, P5, P6, R5, R7, R8, R9, E5, I2, P10, R19, S2**	Impaired Health Condition; Deficit Communication; Impaired mental health condition; Impaired social condition; Trauma exposure; Interpersonal; Preconception; Stressor; Chronic pain
	Life experiences<br>**A3, A8, B1, B2, B4, M2, R9, T2, P11**	Contentment with their lives; Lived Experience; Reminiscences; Memories of a past loss can serve purposes of reconciling with ageing through an affirmation of self as resilient; Effective problem-solving history; Wisdom (wisdom attainment; wisdom that comes from having lived a life); Perceived financial situation

	Physiology factors<br><br>**R12, W2**	Autonomic nervous system, hypothalamic-pituitary axis, innate immunity, renin-angiotensin system, insulin/growth hormone/IGF-1 pathways and their interactions with Genetic, environmental, physiologic, cellular, molecular functions may influence degree of resilience; White matter integrity of the GCC (genu of corpus callosum) and Grit (domain CD-RISC) were association
	Social context<br><br>**B2, B3, C2, C4, D3, G1, H1, I1, M1, P8, R5, R8, R13, U1, U2, W1, A9, B5, E4, H4, P12, R20, T6**	Personal relationships:<br>Social competence; Family as support; Family relations; Family cohesion; Secure relationships; Accord relationships; Social relations; Social connectedness; Religiosity; Participation and social cohesion; Friends support (friends and particularly friends in similar circumstances are a great source of support); Family and friend networks; Social networks; Gerotranscendence<br><br>Environmental support:<br>Material resources; Social support (social policies, employment, neighbourhood, economy, health, social care e other services, rehabilitation, guide dogs, third sector); Environmental systems; Social and welfare services; Social resources (health and social care); Access to formal support; Community resources; Community level; Housing; Peer support; Engagement with art.
	Intrinsic aspects<br><br>**A8, B2, C4, D3, F1, M2, P6, P7, R1, R2, R5, R8, R9, R15, A9, E4, H4, P12, P13**	Have purpose or goals:<br>Capacity building and support provided by case professionals; Moving forward with life; Curiosity/ever-seeking; “Head-On” approach to challenge (study participants expressed the belief that one should make plans when faced with a dilemma and not just sit around waiting for something to happen); Meaningfulness; Maintaining continuity; Having and maintaining interests; Perseverance; Meaning, purpose and growth<br><br>Maintenance balance:<br>Perceived Control; Personal control; Equanimity<br><br>Beliefs:<br>Spirituality and religiosity; Prayer; Spiritual Grounding
	Health conditions<br><br>**C2, C5, D3, M1, M4, F1, H2, P3, P4, P5, P7, R2, R14, T4, W1, A10, C6, D5, H3, P12, R20, S2**	Lifestyle:<br>Independence and autonomy; Performance preserved from activities of daily living; Mobility; Self-care activities; Looking after yourself (self-care); Self-efficacy; "Self-efficacy, outcome expectations and exercise behavior"; Looking after yourself (self-care); Self-preservation; Self-determination; Non-obese body mass; Non-smoker; Satisfaction with quality of sleep; Having a good apetite; Brain fitness; Self-compassion<br><br>Express emotions:<br>Positive emotions; Emotional expression and communication; Emotional regulation; Humor; Hope (low hopelessness scores also predicted greater resilience); Self-esteem; Optimism; Facing fear; Moral compass, ethics, altruism; Cognitive and emotional flexibility; Resignation; Confrontation

	Express self-awareness<br><br>**A3, A8, B2, B1, C4, F1, M2, R2, R5, R8, T3**	Strong sense of identity; Extending self to others; Ceaseless self-improvement; Accept one-self; Accept situation; Maintenance of self-identity; Adaptable personality; Personal competence; Self and personality; Everyday competence; Self-reliance; Self-reflection (self-criticism and the perception of being caregiver or care receiver) the availability of supportive relationships and reciprocity in these relationships as central elements in the development of resilience; "Maverick" (Being mavericks - unconventional); Existential aloneness.
**CONSEQUENTS**	Components of mental health<br><br>**A2, A6, B4, C3, C5, D1, D2, L1, P1, P6, P7, R6, R10, R15, S1, T1, V1, W2, A10, P11, M5, E5**	Control of symptoms psychological:<br>Less depressive symptoms; Less depression; Less apathy; Less anxiety<br><br>Control of emotions:<br>Control of stressful events; Control of stress and negative affect/negative emotion; Stress reduction; Control daily negative emotion; Lower emotional distress; Emotional stability; Express happiness; Maintaining a strong positive self-concept; Express daily positive emotion; Emotional maturity; Gratitude
	Positive perspective and experience of ageing<br><br>**A1, A2, A3, A7, B3, B4, C2, C3, C5, E1, F1, H1, M1, M3, O1, P3, P4, R1, R2, R4, R11, R13, R15, R16, S1, T3, U2, V1, A10, E5, P11, P12, R21, T6, S4, R22, S5, D4, E6, E8, R18, A9, G2, E7, E8, C7, F3, H4, S3**	Active aging:<br>Well-being; Quality of life; Life satisfaction; Successful aging (life satisfaction; morale; health-promoting lifestyle); Self-rated successful ageing; Longevity; Ageing with integrity; Experience an quiet ageing; Savoring life; Being active; Keeping active and busy; Outdoor visits; Walking; Going to churchill<br><br>Independence and autonomy:<br>Different strategies to maintain a sense of independence; Fast gait speed; Keep doing a job and earning a living; Resilience demonstrated more protective for risk of dependence for activities of daily livings in older people (65-84 years); Reduced risk of dependence on activities of daily living; Express stoic attitudes (wisdom and moderation) in facing increasing frailty; Driving abilities<br>Values socialization:<br>Greater social participation; Greater perception social support; Maintain active social (to value intergenerationality and relationship with youngest); Value interaction with friends, family and neighbours; Volunteering<br><br>Personal behavior:<br>Personal growth; Purpose in life; Continuity; Perseverance; Self-acceptance; Redemption; Acquiring an empowering perspective; Learning to let go and moving on'; Remaining active and keeping busy; Individual innovativeness; Sense Purpose; Religiosity; Spirituality

	Grief and loss experience<br><br>**B2, D1, P9, R14, E4**	Recovery with loss; Protection factor against widowhood; They lived active lives, indicating a relatively stable, healthy level of psychological, and physical functioning following the death of their spouse; do not express denial or avoid talking about the loss
	Coping strategies<br><br>**A1, A8, B4, E1, H1, M4, R1, R4, O1, V1, E7, F2, N3, R19**	Problem-solving; Coping escape (avoids stressful situations); Practical coping; Emotional coping; Spiritual coping; Problem coping; Emotional coping; Coping styles; Coping resources; Courage and resourcefulness; Speaking it out was expressed as a strategy to relieve hardship; Successfully coping with adversity; Coping with unfavorable situations, recovery and/or maintenance of health, personal and family protection; Strategy of coping with vulnerabilities; Strategy of coping with discrimination; Coping to financial hardship; Coping to nutrition risk
	Health perspective<br><br>**A4, H2, I1, L1, P9, T1, R7, R12, R14, P14, E9**	Positive health self-report; Good physical and mental health; Less impact related to the treatment of chronic and acute conditions; Expresses homeostasis conditions and basal levels
	Optimistic perspective<br><br>**A6, C1, D3, F1, M1, R2, P11, E4, P12, R18, R21**	Optimism; Maintaining a positive outlook on life; Positive reappraisal; Positive Attitude; Express positive and being thankful for life; Happiness; Good humor
**EMPIRICAL ELEMENTS**	Scales<br><br>**A2, A4, A7, C1, C5, H2, M1, N2, P1, P2, P8, R6, R7, V1, W2, M2, I2, R11, B1, R9, T1, T4, E2, D1, A1, R4, T2, P6, P7, P8, B2, D2, S1, P7, L1, C6, E7, P11, P12, R19, R20, S3, S5, A9, E4, E6, F3, G2, P12, S2, F2, P14, A10, B5, C7, H4, N3, P10, R17, H3, D4, D6, P13**	Connor Davidson Resilience Scale (Four domains - grit, active coping self-efficacy, accommodative coping self-efficacy e spirituality); The Resilience Scale (Two domains - personal competence; acceptance of self and life); Simplified Resilience Score=Leave Behind Questionnaire + Resilience Scale; Brief Resilient Coping Scale; Dispositional Resilience Scale; Groningen Ageing Resilience Inventory; The Hardy-Gill Resilience Scale; Resilience in Older Adults Survey; The Ego-Resilience Scale; John Henry Active Coping level; Psychological Resilience Scale for Adults; Psychological Resilience Against Physical Difficulties Index (PRAPDI).

	Interventions and qualitative assessment<br><br>**A3, A5, B3, C2, C3, C4, E1, F1, H1, M3, N1, P3, R2, P4, R3, R5, R13, R15, T3, U1, W1, E4, R21, T6, T5, R21, R18, F2, E8, E4**	To explore significant life events and experiences; To explore events of adversity and confrontation; To explore resilience; To explore support mechanisms; To measure social support; To measure self-efficacy; To measure self-awareness; To explore physical and cognitive performance; To explore how is it getting old; To explore resilient characteristics.<br><br>In context of the COVID-19 Pandemic: To explore challenges experienced; How COVID-19 affected their lives; What the changes in that period were; To explore social networks and assistance from government agencies or communities during the pandemic; To explore impact financial hardship during COVID-19; To explore strategies, resources and processes do older adults.

A1 Tomás, J. M.; Meléndez, J. C.; Sancho, P.; Mayordomo, T. Adaptation and initial validation of the BRCS in an elderly spanish sample. *Eur J Psychol Assess* **2012,** 28(4), 283–289. https://doi.org/10.1027/1015-5759/a000108

A2 Jeste, D. V.; Savla, G. N.; Thompson, W. K.; Vahia, I. V.; Glorioso, D. K.; Martin, A. S.; Palmer, B. W.; Rock, D.; Golshan, S.; Kraemer, H. C.; Depp, C. A. Association between older age and more successful aging: critical role of resilience and depression. *Am J Psychiatry* **2013**, 170(2), 188–196. https://doi.org/10.1176/appi.ajp.2012.12030386

A3 Browne-Yung, K.; Walker, R. B.; Luszcz, M. A. An examination of resilience and coping in the oldest old using life narrative method. *Gerontologist*, **2015,** 57(2), 282-291. https://doi.org/10.1093/geront/gnv137

A4 Zhang, K.; Zhang, W.; Wu, B.; Liu, S. Anxiety about aging, resilience and health status among Chinese older adults: findings from Honolulu and Wuhan. *Arch Gerontol Geriatr,* **2020,** 104015. https://doi.org/10.1016/j.archger.2020.104015

A5 Browne, C. V.; Mokuau, N.; Braun, K. L. Adversity and resiliency in the lives of native Hawaiian elders. *Social Work* **2009,** 54(3), 253–261. https://doi.org/10.1093/sw/54.3.253

A6 Harris, P. B. Another wrinkle in the debate about successful aging: the undervalued concept of resilience and the lived experience of dementia. *Int J Aging Hum Dev,* **2008,** 67(1), 43–61. https://doi.org/10.2190/ag.67.1.c

A7 Levasseur, M.; Roy, M.; Michallet, B.; St-Hilaire, F.; Maltais, D.; Généreux, M. Associations between resilience, community belonging, and social participation among community-dwelling older adults: results from the eastern townships population health survey. *Arch Phys Med Rehabil* **2017,** 98(12), 2422–2432. https://doi.org/10.1016/j.apmr.2017.03.025

A8 Wagnild, G. M.; Collins, J. A. Assessing Resilience. *J Psychosoc Nurs Ment Health Serv* **2009,** 47(12), 28–33. https://doi.org/10.3928/02793695-20091103-01

A9 Ferreira, G. S.; Souto, R. Q.; Almeida, S. A. de; Ribeiro, G. D. S.; Araújo, G. K. N. de; Santos, R. D. C. Assessment of resilience profile and associated factors in community elderly. *Rev. enferm. UERJ* **2020,** 28, e51659. https://doi.org/10.12957/reuerj.2020.51659

A10 Perez-Rojo, G.; López, J.; Noriega, C.; Velasco, C.; Carretero, I.; López-Frutos, P.; Galarraga, L. A multidimensional approach to the resilience in older adults despite COVID-19. *BMC Geriatrics,* **2022,** 22(1). https://doi.org/10.1186/s12877-022-03472-y

B1 Ohana, I.; Golander, H.; Barak, Y. Balancing psychache and resilience in aging Holocaust survivors. *Int Psychogeriatr* **2014,** 26(6), 929–934. https://doi.org/10.1017/s104161021400012x

B2 Van Abbema, R.; Bielderman, A.; de Greef, M.; Hobbelen, H.; Krijnen, W.; Van der Schans, C. Building from a conceptual model of the resilience process during ageing, towards the Groningen Aging Resilience Inventory. *J Adv Nurs* **2015,** 71(9), 2208–2219. https://doi.org/10.1111/jan.12685

B3 Higgins, A.; Sharek, D.; Glacken, M. Building resilience in the face of adversity: navigation processes used by older lesbian, gay, bisexual and transgender adults living in Ireland. *JCN* **2016,** 25(23-24), 3652–3664. https://doi.org/10.1111/jocn.13288

B4 Gattuso, S. Becoming a wise old woman: resilience and wellness in later life. *Health Sociol. Rev.* **2003,** 12(2), 171–177. https://doi.org/10.5172/hesr.12.2.171

B5 da Silva-Sauer, L.; de la Torre-Luque, A.; Smith, B. W.; C. M. C. Lins, M.; Andrade, S.; Fernández-Calvo, B. Brief Resilience Scale (BRS) Portuguese Version: validity and metrics for the older adult population. *Aging Ment Health* **2020,** 1–10. https://doi.org/10.1080/13607863.2020.1753015

C1 Rana, B. K.; Darst, B. F.; Bloss, C.; Shih, P. B.; Depp, C.; Nievergelt, C. M.; Allison, M.; Parsons, J. K.; Schork, N.; Jeste, D. V. Candidate SNP associations of optimism and resilience in older adults: exploratory study of 935 community-dwelling adults. *Am J Geriatr Psychiatry* **2014,** 22(10), 997-1006.e5. https://doi.org/10.1016/j.jagp.2014.03.009

C2 Li, J.; Xu, L.; Chi, I. Challenges and resilience related to aging in the United States among older Chinese immigrants. *Aging Ment Health* **2017**, 22(12), 1548–1555. https://doi.org/10.1080/13607863.2017.1377686

C3 Maercker, A., Hilpert, P., Burri, A. Childhood trauma and resilience in old age: applying a context model of resilience to a sample of former indentured child laborers. *Aging Ment Health* **2015,** 20(6), 616–626. https://doi.org/10.1080/13607863.2015.1033677

C4 Lou, V. W. Q.; Ng, J. W. Chinese older adults' resilience to the loneliness of living alone: a qualitative study. *Aging Ment Health* **2012,** 16(8), 1039–1046. https://doi.org/10.1080/13607863.2012.692764

C5 Laird, K. T.; Lavretsky, H.; Paholpak, P.; Vlasova, R. M.; Roman, M.; Cyr, N. S.; Siddarth, P. Clinical correlates of resilience factors in geriatric depression. *Int Psychogeriatr* **2019,** 31(2), 193–202. https://doi.org/10.1017/S1041610217002873

C6 Carriedo, A.; Cecchini, P. J. A.; Fernandez-Rio, J.; Méndez-Giménez, A. COVID-19, psychological well-being and physical activity levels in older adults during the nationwide lockdown in Spain. *Am J Geriatr Psychiatry* **2020,** 28(11). https://doi.org/10.1016/j.jagp.2020.08.007

C7 Silva-Sauer, L.; Martins-Rodrigues, R.; Torre-Luque, A.; Fernández-Calvo, B. Cross-cultural adaptation and psychometric properties of the Brazilian Portuguese version of successful aging scale in community-dwelling older adults. *J Community Psychol* **2020** https://doi.org/10.1002/jcop.22374

D1 King, B. M.; Carr, D. C.; Taylor, M. G. Depressive symptoms and the buffering effect of resilience on widowhood by gender. *Gerontologist* **2018,** https://doi.org/10.1093/geront/gny115

D2 Mehta, M.; Whyte, E.; Lenze, E.; Hardy, S.; Roumani, Y.; Subashan, P.; Huang, W.; Studenski, S. Depressive symptoms in late life: associations with apathy, resilience and disability vary between young-old and old-old. *Int J Geriatr Psychiatry* **2008**, 23(3), 238–243. https://doi.org/10.1002/gps.1868

D3 Martin, A. S.; Distelberg, B.; Palmer, B. W., & Jeste, D. V. Development of a new multidimensional individual and interpersonal resilience measure for older adults. *Aging Ment Health* **2014,** 19(1), 32–45. https://doi.org/10.1080/13607863.2014.909383

D4 Kemer, A. S.; Gökalp, K. Determination of the effects of innovativeness levels of the elderly on their psychological resilience. *Perspect Psychiatr Care* **2020.** https://doi.org/10.1111/ppc.12629

D5 Pedone, C.; Costanzo, L.; Finamore, P.; Bandinelli, S.; Ferrucci, L.; Antonelli Incalzi, R. Defining resilience in older people: does a subjective definition of stressor work? *J Gerontol A Biol Sci Med Sci.* **2020** https://doi.org/10.1093/gerona/glaa189

D6 Li, Y.-T.; Ow, Y. S. Y. Development of resilience scale for older adults. *Aging Ment Health* **2021,** 26:1, 159-158. https://doi.org/10.1080/13607863.2020.1861212

E1 Manning, L. K. Enduring as lived experience: exploring the essence of spiritual resilience for women in late life. *J Relig Health* **2012,** 53(2), 352–362. https://doi.org/10.1007/s10943-012-9633-6

E2 Windle, G.; Markland, D. A.; Woods, R. T. Examination of a theoretical model of psychological resilience in older age. *Aging Ment Health* **2008**, 12(3), 285–292. https://doi.org/10.1080/13607860802120763

E3 Hildon, Z.; Smith, G.; Netuveli, G.; Blane, D. Understanding adversity and resilience at older ages. *Sociol Health Illn* **2008**, 30(5), 726–740. https://doi.org/10.1111/j.1467-9566.2008.01087.x

E4 Shen, S.-F.; Lee, S.-Y.; Tung, H.-H.; Hsu, S.-F. Effect of negative life events and resilience on health status of older adults with possible sarcopenia: a qualitative research. *Int J Gerontol* **2021**, 15, 367–371. https://doi.org/1%20https://doi.org/10.6890/IJGE.202110_15(4).0014

E5 Manning, L. K.; Bouchard, L. Encounters with adversity: a framework for understanding resilience in later life. *Aging Ment Health* **2019,** 24(7), 1–8. https://doi.org/10.1080/13607863.2019.1594162

E6 St. Louis, R. M.; Koppel, S.; Molnar, L. J.; Di Stefano, M.; Darzins, P.; Porter, M. M.; Bédard, M.; Mullen, N.; Myers, A.; Marshall, S.; Charlton, J. L. Examining the contribution of psychological resilience on self-reported and naturalistic driving behavior of older adults. *J Safety Res* **2022,** 82, 251–260. https://doi.org/10.1016/j.jsr.2022.06.001

E7 Mao, W.; Wu, B.; Chi, I.; Yang, W.; Dong, X. Experiences of discrimination and oral health-related quality of life among foreign-born older Chinese Americans: does resilience play a mediating role? *Community Dent Oral Epidemiol* **2021.** https://doi.org/10.1111/cdoe.12723

E8 Lee, K.; Hyun, K.; Mitchell, J.; Saha, T.; Oran Gibson, N.; Krejci, C. Exploring factors enhancing resilience among marginalized older adults during the COVID-19 pandemic. *J Appl Gerontol,* **2021,** 073346482110487. https://doi.org/10.1177/07334648211048749

F1 Ottmann, G.; Maragoudaki, M. Fostering resilience later in life: a narrative approach involving people facing disabling circumstances, carers and members of minority groups. *Ageing Soc* **2014,** 35(10), 2071–2099. https://doi.org/10.1017/s0144686x14000828

F2 Taylor, M. G.; Carr, D. C.; Jason, K. Financial hardship and psychological resilience during COVID-19: differences by race/ethnicity. *J Gerontol B Psychol Sci Soc Sci* **2021.** https://doi.org/10.1093/geronb/gbab173

F3 Salamene, L. C.; Martins, E. L. M.; Lucchetti, G.; Lucchetti, A. L. G. Factors associated with successful aging in Brazilian community-dwelling older adults: when physical health is not enough. *Geriatr Nurs* **2021,** 42(2), 372–378. https://doi.org/10.1016/j.gerinurse.2021.01.009

G1 Yee-Melichar, D.; Boyle, A. R.; Wanek, L. J.; Pawlowsky, S. B. Geriatric rehabilitation and resilience from a cultural perspective. *Geriatr Nurs* **2014,** 35(6), 451-454.e9. https://doi.org/10.1016/j.gerinurse.2014.08.010

G2 Mendoza, A. N.; Fruhauf, C. A.; MacPhee, D. Grandparent caregivers' resilience: stress, support, and coping predict life satisfaction. *Int J Aging Hum Dev* **2019,** 91(1), 3–20. https://doi.org/10.1177/0091415019843459

H1 Kwong, K.; Du, Y.; Xu, Q. Healthy aging of minority and immigrant populations: resilience in late life. *Traumatology* **2015**, 21(3), 136–144. https://doi.org/10.1037/trm0000034

H2 McKibbin, C.; Lee, A.; Steinman, B. A.; Carrico, C.; Bourassa, K.; Slosser, A. Health status and social networks as predictors of resilience in older adults residing in rural and remote environments. *J Aging Res* **2016,** https://doi.org/10.1155/2016/4305894

H3 Wister, A.; Cosco, T.; Mitchell, B.; Fyffe, I; Health behaviors and multimorbidity resilience among older adults using the Canadian Longitudinal Study on Aging. *International Psychogeriatrics,* **2019,** 1–15. https://doi.org/10.1017/s1041610219000486

H4 Gallardo-Peralta, L. P.; Mayorga Muñoz, C.; Soto Higuera, A. Health, social support, resilience and successful ageing among older Chilean adults. *Int Soc Work* **2020,** 65:2, 283-299 https://doi.org/10.1177/0020872819901147

I1 Mhaka-Mutepfa, M.; Mpofu, E.; Cumming, R. Impact of protective factors on resilience of grandparent carers fostering orphans and non-orphans in Zimbabwe. *J Aging Health* **2014,** 27(3), 454–479. https://doi.org/10.1177/0898264314551333.

I2 Chhetri, J. K.; Xue, Q. L.; Ma, L.; Chan, P.; Varadhan, R. Intrinsic capacity as a determinant of physical resilience in older adults. *J Nutr Health Aging* **2021,** https://doi.org/10.1007/s12603-021-1629-z

L1 Gupta, S.; Bélanger, E.; Phillips, S. P. Low socioeconomic status but resilient: panacea or double trouble? John Henryism in the International IMIAS study of older adults. *J Cross Cult Gerontol* **2018,** 34(1), 15–24. https://doi.org/10.1007/s10823-018-9362-8

M1 Lamond, A. J.; Depp, C. A.; Allison, M.; Langer, R.; Reichstadt, J.; Moore, D. J.; Golshan, S.; Ganiats, T. G.; Jeste, D. V. Measurement and predictors of resilience among community-dwelling older women. *J Psychiatr Res* **2008,** 43(2), 148–154. https://doi.org/10.1016/j.jpsychires.2008.03.007

M2 Yang, F.; Bao, J. M.; Huang, X. H.; Guo, Q.; Smith, G. D. Measurement of resilience in Chinese older people. *Int Nurs Rev* **2015,** 62(1), 130–139. https://doi.org/10.1111/inr.12168

M3 Koren, C. Men's vulnerability–women's resilience: from widowhood to late-life repartnering. *Int Psychogeriatr* **2015,** 28(5), 719–731. https://doi.org/10.1017/s1041610215002240

M4 Pathike, W.; O'Brien, A. P.; Hunter, S. Moving on from adversity: an understanding of resilience in rural Thai older people. *Aging Ment Health* **2017,** 23(3), 311–318. https://doi.org/10.1080/13607863.2017.1411883

M5 Wister, A.; Li, L.; Cosco, T. D.; McMillan, J.; Griffith, L. E.; Costa, A.; Anderson, L.; Balion, C.; Kirkland, S.; Yukiko, A.; Wolfson, C.; Basta, N.; Cossette, B.; Levasseur, M.; Hofer, S.; Paterson, T.; Hogan, D.; Liu-Ambrose, T.; Menec, V.; St. John, P. Multimorbidity resilience and COVID-19 pandemic self-reported impact and worry among older adults: a study based on the Canadian Longitudinal Study on Aging (CLSA). *BMC Geriatrics* **2022,** 22(1). https://doi.org/10.1186/s12877-022-02769-2

M6 Wister, A.; Cosco, T.; Mitchell, B.; Fyffe, I. Health behaviors and multimorbidity resilience among older adults using the Canadian Longitudinal Study on Aging. *Int Psychogeriatr* **2019,** 1–15. https://doi.org/10.1017/s1041610219000486

N1 Randall, W.; Baldwin, C.; McKenzie-Mohr, S.; McKim, E.; Furlong, D. Narrative and resilience: a comparative analysis of how older adults story their lives. *J Aging Stud* **2015,** 34, 155–161. https://doi.org/10.1016/j.jaging.2015.02.010

N2 Laird, K. T.; Lavretsky, H.; St. Cyr, N.; Siddarth, P. Resilience predicts remission in antidepressant treatment of geriatric depression. *Int J Geriatr Psychiatry* **2018**, 33(12), 1596–1603. https://doi.org/10.1002/gps.4953

N3 Capicio, M.; Panesar, S.; Keller, H.; Gramlich, L.; Popeski, N.; Basualdo-Hammond, C.; Atkins, M.; Chan, C. B. Nutrition risk, resilience and effects of a brief education intervention among community-dwelling older adults during the COVID-19 pandemic in Alberta, Canada. *Nutrients* **2022**, 14(5), 1110. https://doi.org/10.3390/nu14051110

O1 Moyle, W.; Clarke, C.; Gracia, N.; Reed, J.; Cook, G.; Klein, B.; Marais, S.; Richardson, E. Older people maintaining mental health well-being through resilience: an appreciative inquiry study in four countries. *J Nurs Healthc Chronic Illn* **2010**, 2(2), 113–121. https://doi.org/10.1111/j.1752-9824.2010.01050.x

P1 Zhong, X.; Wu, D.; Nie, X.; Xia, J.; Li, M.; Lei, F.; Lim, H. A.; Kua, E.-H.; Mahendran, R. Parenting style, resilience, and mental health of community-dwelling elderly adults in China. *BMC Geriatrics,* **2016,** 16(1). https://doi.org/10.1186/s12877-016-0308-0

P2 Kohler, S.; Loh, S. M. Patient resilience in the fracture orthopaedic rehabilitation geriatric environment. *Australas J Ageing* **2016,** 36(1), 65–68. https://doi.org/10.1111/ajag.12362

P3 Ribeiro, M. dos S.; Borges, M. da S. Perceptions of aging and falling ill: a study with elderly persons in palliative care. *Revista Brasileira de Geriatria e Gerontologia* **2018,** 21(6), 701–710. https://doi.org/10.1590/1981-22562018021.180139

P4 Yang, Y.; Wen, M. Psychological resilience and the onset of activity of daily living disability among older adults in China: a nationwide longitudinal analysis. *J Gerontol B Psychol Sci Soc Sci* **2014,** 70(3), 470–480. https://doi.org/10.1093/geronb/gbu068

P5 Gooding, P. A.; Hurst, A.; Johnson, J.; Tarrier, N. Psychological resilience in young and older adults. *Int J Geriatr Psychiatry* **2011,** 27(3), 262–270. https://doi.org/10.1002/gps.2712

P6 O'Rourke, N.; Kupferschmidt, A. L.; Claxton, A.; Smith, J. Z.; Chappell, N.; Beattie, B. L. Psychological resilience predicts depressive symptoms among spouses of persons with Alzheimer disease over time. *Aging Ment Health* **2010,** 14(8), 984–993. https://doi.org/10.1080/13607863.2010.501063

P7 Ong, A. D.; Bergeman, C. S.; Bisconti, T. L.; Wallace, K. A. Psychological resilience, positive emotions, and successful adaptation to stress in later life. *J Pers Soc Psychol* **2006,** 91(4), 730–749. https://doi.org/10.1037/0022-3514.91.4.730

P8 Diamond, S.; Shrira, A. Psychological vulnerability and resilience of Holocaust survivors engaged in creative art. *Psychiatry Research* **2018** 264, 236–243. https://doi.org/10.1016/j.psychres.2018.04.013

P9 Bonanno, G. A.; Wortman, C. B.; Nesse, R. M. Prospective patterns of resilience and maladjustment during widowhood. *Psychol Aging* **2004**, 19(2), 260–271. https://doi.org/10.1037/0882-7974.19.2.260

P10 Zaidel, C.; Musich, S.; Karl, J.; Kraemer, S.; Yeh, C. S. Psychosocial factors associated with sleep quality and duration among older adults with chronic pain. *Popul Health Manag* **2020.** https://doi.org/10.1089/pop.2019.0165

P11 Tourunen, A.; Siltanen, S.; Saajanaho, M.; Koivunen, K.; Kokko, K.; Rantanen, T. Psychometric properties of the 10-item Connor–Davidson resilience scale among finnish older adults. *Aging Ment Health,* **2019** 1–8. https://doi.org/10.1080/13607863.2019.1683812

P12 Kusz, H.; Ahmad, A. Preserving engagement, nurturing resilience. *Clin Geriatr Med* **2020.** https://doi.org/10.1016/j.cger.2020.06.004

P13 Asch, R. H.; Kachadourian, L.; Southwick, S. M.; Esterlis, I.; Pietrzak, R. H. Psychological resilience to the challenges of physical aging in older U.S. veterans: results from the 2019-2020 National Health and Resilience in Veterans Study. *Am J Geriatr Psychiatry* **2021** https://doi.org/10.1016/j.jagp.2021.04.013

P14 Taylor, M. G.; Carr, D. Psychological resilience and health among older adults: a comparison of personal resources. *J Gerontol B Psychol Sci Soc Sci.* **2020** https://doi.org/10.1093/geronb/gbaa116

R1 Reis, L. A. dos; Menezes, T. M. de O. Religiosity and spirituality as resilience strategies among long-living older adults in their daily lives. *Rev Bras Enferm*, **2017** 70(4), 761–766. https://doi.org/10.1590/0034-7167-2016-0630

R2 Wagnild, G.; Young, H. M. Resilience among older women. *J Nurs Scholarsh,* **1990,** 22(4), 252–255. https://doi.org/10.1111/j.1547-5069.1990.tb00224.x

R3 Bennett, K. M.; Reyes-Rodriguez, M. F.; Altamar, P.; Soulsby, L. K. Resilience amongst older colombians living in poverty: an ecological approach. *J Cross Cult Gerontol,* **2016,** 31(4), 385–407. https://doi.org/10.1007/s10823-016-9303-3

R4 Tomás, J. M.; Sancho, P.; Melendez, J. C.; Mayordomo, T. Resilience and coping as predictors of general well-being in the elderly: a structural equation modeling approach. *Aging Ment Health,* **2012** *16*(3), 317–326. https://doi.org/10.1080/13607863.2011.615737

R5 Kinsel, B. Resilience as adaptation in older women. *J Women Aging* **2005,** 17(3), 23–39. https://doi.org/10.1300/j074v17n03_03

R6 Laird, K. T.; Lavretsky, H.; St. Cyr, N.; Siddarth, P. Resilience predicts remission in antidepressant treatment of geriatric depression. *Int J Geriatr Psychiatry,* **2018** 33(12), 1596–1603. https://doi.org/10.1002/gps.4953

R7 Smith, P. R. Resilience: resistance factor for depressive symptom. *J Psychiatr Ment Health Nurs* **2009** 16(9), 829–837. https://doi.org/10.1111/j.1365-2850.2009.01463.x

R8 Montpetit, M. A.; Bergeman, C. S.; Deboeck, P. R.; Tiberio, S. S.; Boker, S. M. Resilience as process: negative affect, stress, and coupled dynamical systems. *Psychology and Aging,* **2010,** 25(3), 631–640. https://doi.org/10.1037/a0019268

R9 Nygren, B.; Aléx, L.; Jonsén, E.; Gustafson, Y.; Norberg, A.; Lundman, B. Resilience, sense of coherence, purpose in life and self-transcendence in relation to perceived physical and mental health among the oldest old. *Aging Ment Health* **2005** (4), 354–362. https://doi.org/10.1080/1360500114415

R10 Lim, M. L.; Lim, D.; Gwee, X.; Nyunt, M. S. Z.; Kumar, R.; Ng, T. P. Resilience, stressful life events, and depressive symptomatology among older Chinese adults. *Aging Ment Health* **2015** 19(11), 1005–1014. https://doi.org/10.1080/13607863.2014.995591

R11 Wells, M.; Avers, D.; Brooks, G. Resilience, physical performance measures, and self-perceived physical and mental health in older catholic nuns. *J Geriatr Phys Ther* **2012** 35(3), 126–131. https://doi.org/10.1519/jpt.0b013e318237103f

R12 Hadley, E. C.; Kuchel, G. A.; Newman, A. B.; Allore, H. G.; Bartley, J. M.; Bergeman, C. S.; Blinov, M. L.; Colon-Emeric, C. S.; Dabhar, F. S.; Dugan, L. L.; Dutta, C.; Eldadah, B. A.; Ferrucci, L.; Kirkland, J. L.; Kritchevsky, S. B.; Lipsitz, L. A.; Nadkarni, N. K.; Reed, M. J.; Schmader, K. E.; Sierra, F. Report: NIA Workshop on measures of physiologic resiliencies in human aging. *J Gerontol A Biol Sci Med Sci* **2017** 72(7), 980–990. https://doi.org/10.1093/gerona/glx015

R13 Thetford, C.; Bennett, K. M.; Hodge, S.; Knox, P. C.; Robinson, J. Resilience and vision impairment in older people. *J Aging Stud* **2015** 35, 37–48. https://doi.org/10.1016/j.jaging.2015.07.007

R14 Resnick, B. Resilience in older adults. *Top Geriatr Rehabil* **2014,** 30(3), 155–163. https://doi.org/10.1097/tgr.0000000000000024

R15 Diehl, M.; Hay, E. L. Risk and resilience factors in coping with daily stress in adulthood: the role of age, self-concept incoherence, and personal control. *Developmental Psychology* **2010,** 46(5), 1132–1146. https://doi.org/10.1037/a0019937

R16 Wagnild, G. Resilience and successful aging: comparison among low and high income older adults. *J Gerontol Nurs* **2003,** 29(12), 42–49. https://doi.org/10.3928/0098-9134-20031201-09

R17 Silva-Sauer, L.; Basso Garcia, R.; Pereira da Silva, T. M.; Barbosa de Melo, C.; Fernández-Calvo, B. Relationship between psychological resilience, perceived stress, depression, and physical health in community-dwelling older adults. *Psychol Neurosci* **2021,** 14(2), 132–144. https://doi.org/10.1037/pne0000254

R18 Herron, R. V.; Lawrence, B. C.; Newall, N. E. G.; Ramsey, D.; Waddell- Henowitch, C. M.; Dauphinais, J. Rural older adults' resilience in the context of COVID-19. *Soc. sci. med,* **2022,** 306, 115153. https://doi.org/10.1016/j.socscimed.2022.115153

R19 Zapater-Fajarí, M.; Crespo-Sanmiguel, I.; Pulopulos, M. M.; Hidalgo, V.; Salvador, A. Resilience and psychobiological response to stress in older people: the mediating role of coping strategies. *Front Aging Neurosci* **2021,** 13. https://doi.org/10.3389/fnagi.2021.632141

R20 Liu, Z.; Zhou, X.; Zhang, W.; Zhou, L. Resilience and its correlates among first ischemic stroke survivors at acute stage of hospitalization from a tertiary hospital in China: a cross-sectional study. *Aging Ment Health* **2018**, 24(5), 828–836. https://doi.org/10.1080/13607863.2018.1550630

R21 Silva Júnior, E. G. da; Eulálio, M. do C. Resiliência para uma velhice bem-sucedida: mecanismos sociais e recursos pessoais de proteção. *Psicologia: ciência e profissão* **2022,** 42. https://doi.org/10.1590/1982-3703003234261

R22 Cesari, M.; Azzolino, D.; LeBrasseur, N. K.; Whitson, H.; Rooks, D.; Sourdet, S.; Angioni, D.; Fielding, R. A.; Vellas, B.; Rolland, Y. Resilience: biological basis and clinical significance - a perspective report from the International Conference on Frailty and Sarcopenia Research (ICFSR) Task Force. *J Frailty Aging.* **2022** https://doi.org/10.14283/jfa.2022.62

S1 Smith, J. L.; Hollinger-Smith, L. Savoring, resilience, and psychological well-being in older adults. *Aging Ment Health* **2014,** 19(3), 192–200. https://doi.org/10.1080/13607863.2014.986647

S2 Höltge, J.; Mc Gee, S. L.; Maercker, A.; Thoma, M. V. Steeling in later life: exploring age-specific effects of varying levels of stress on psychological resilience. *Int J Aging Hum Dev* **2019,** https://doi.org/10.1177/0091415019871202

S3 Qi, X.; Zhang, W.; Wang, K.; Pei, Y.; Wu, B. Social isolation and psychological well-being among older chinese americans: does resilience mediate the association? *Int J Geriatr Psychiatry* **2022,** 37(8). https://doi.org/10.1002/gps.5791

S4 Whitmore, C.; Markle-Reid, M.; McAiney, C.; Ploeg, J.; Griffith, L. E.; Phillips, S. P.; Wister, A.; Fisher, K. Self-reported health and the well-being paradox among community-dwelling older adults: a cross-sectional study using baseline data from the Canadian Longitudinal Study on Aging (CLSA). *BMC Geriatrics 2022,* 22(1). https://doi.org/10.1186/s12877-022-02807-z

S5 Siltanen, S.; Portegijs, E.; Saajanaho, M.; Pynnönen, K.; Kokko, K.; Rantanen, T. Self-rated resilience and mobility limitations as predictors of change in active aging during COVID-19 restrictions in Finland: a longitudinal study. *Eur J Ageing* **2021,** 19(3), 475–484. https://doi.org/10.1007/s10433-021-00634-6

T1 Cohen, M.; Baziliansky, S.; Beny, A. The association of resilience and age in individuals with colorectal cancer: an exploratory cross-sectional study. *J Geriatr Oncol* **2014,** 5(1), 33–39. https://doi.org/10.1016/j.jgo.2013.07.009

T2 Meléndez, J. C.; Fortuna, F. B.; Sales, A.; Mayordomo, T. The effects of instrumental reminiscence on resilience and coping in elderly. Archives of Gerontology and Geriatrics **2015,** 60(2), 294–298. https://doi.org/10.1016/j.archger.2014.12.001

T3 Metze, R. N., Kwekkeboom, R. H., & Abma, T. A. (2015). The potential of family group conferencing for the resilience and relational autonomy of older adults. *Journal of Aging Studies,* 34, 68–81. https://doi.org/10.1016/j.jaging.2015.04.005

T4 Resnick, B. A., & Inguito, P. L. (2011). The Resilience Scale: psychometric properties and clinical applicability in older adults. *Archives of Psychiatric Nursing,* 25(1), 11–20. https://doi.org/10.1016/j.apnu.2010.05.001

T5 Abulaiti, B., Zhang, X., Guan, T., Wang, M., Jia, S., & Wang, A. (2022). The dyadic care experiences of elderly individuals with disabilities and caregivers in the home setting from the perspective of family resilience: a qualitative study. *Frontiers in Psychiatry, 13*. https://doi.org/10.3389/fpsyt.2022.963101

T6 Klokgieters, S. S., van Tilburg, T. G., Deeg, D. J. H., & Huisman, M. (2018). The linkage between ageing, migration and resilience: resilience among young-old Turkish and Moroccan immigrants. *Innovation in Aging,* 2(suppl1), 70–70. https://doi.org/10.1093/geroni/igy023.262

U1 Hildon, Z., Smith, G., Netuveli, G., & Blane, D. (2008). Understanding adversity and resilience at older ages. *Sociology of Health & Illness,* 30(5), 726–740. https://doi.org/10.1111/j.1467-9566.2008.01087.x

U2 Palma-Candia, O., Hueso Montoro, C., Martí-García, C., Fernández-Alcántara, M., Campos-Calderón, C. P., & Montoya Juárez, R. (2019). Understanding the occupational adaptation process and well-being of older adults in Magallanes (Chile): a qualitative study. *International Journal of Environmental Research and Public Health,* 16(19). https://doi.org/10.3390/ijerph16193640

V1 Dolores Serrano-Parra, M.; Garrido-Abejar, M.; Notario-Pacheco, B.; Bartolomé-Gutiérrez, R.; Solera-Martínez, M.; Martínez-Vizcaíno, V. Validez de la escala de resiliencia de Connor-Davidson (10 ítems) en una población de mayores no institucionalizados. *Enferm Clin* **2013,** 23(1), 14–21. https://doi.org/10.1016/j.enfcli.2012.11.006

W1 Donnellan, W. J.; Bennett, K. M.; Soulsby, L. K. What are the factors that facilitate or hinder resilience in older spousal dementia carers? A qualitative study. *Aging Ment Health* **2014,** 19(10), 932–939. https://doi.org/10.1080/13607863.2014.977771

W2 Krause, B.; Vlasova, R.; Leaver, A.; Laird, K. T.; Siddarth, P.; Narr, K.; Lavretsky, H. White matter integrity in the corpus callosum is associated with resilience factors in geriatric depression. *Am J Geriatr Psychiatry* **2018**, 26(3), S117. https://doi.org/10.1016/j.jagp.2018.01.142
